# Analysis of cerebrospinal fluid metabolites in patients with primary or metastatic central nervous system tumors

**DOI:** 10.1186/s40478-018-0588-z

**Published:** 2018-08-31

**Authors:** Leomar Y. Ballester, Guangrong Lu, Soheil Zorofchian, Venkatrao Vantaku, Vasanta Putluri, Yuanqing Yan, Octavio Arevalo, Ping Zhu, Roy F. Riascos, Arun Sreekumar, Yoshua Esquenazi, Nagireddy Putluri, Jay-Jiguang Zhu

**Affiliations:** 1 Department of Pathology and Laboratory Medicine, University of Texas Health Science Center at Houston, 6431 Fannin St., MSB 2.136, Houston, TX 77030 USA; 2 Department of Neurosurgery, University of Texas Health Science Center at Houston, 6431 Fannin St., MSB 2.136, Houston, TX 77030 USA; 3 Department of Radiology, University of Texas Health Science Center at Houston, Houston, TX 77030 USA; 4 Department of Molecular and Cellular Biology, Baylor College of Medicine, 120D, Jewish Building, One Baylor Plaza, Houston, TX 77030 USA; 5 Advanced Technology Core, Baylor College of Medicine, Houston, TX 77030 USA; 6 Memorial Hermann Hospital, Houston, TX 77030 USA

**Keywords:** CSF, Metabolites, Brain tumor, Glioma, Hydroxyglutarate, Liquid biopsy

## Abstract

Cancer cells have altered cellular metabolism. Mutations in genes associated with key metabolic pathways (e.g., isocitrate dehydrogenase 1 and 2, *IDH1/IDH2*) are important drivers of cancer, including central nervous system (CNS) tumors. Therefore, we hypothesized that the abnormal metabolic state of CNS cancer cells leads to abnormal levels of metabolites in the CSF, and different CNS cancer types are associated with specific changes in the levels of CSF metabolites. To test this hypothesis, we used mass spectrometry to analyze 129 distinct metabolites in CSF samples from patients without a history of cancer (*n* = 8) and with a variety of CNS tumor types (*n* = 23) (i.e., glioma IDH-mutant, glioma-IDH wildtype, metastatic lung cancer and metastatic breast cancer). Unsupervised hierarchical clustering analysis shows tumor-specific metabolic signatures that facilitate differentiation of tumor type from CSF analysis. We identified differences in the abundance of 43 metabolites between CSF from control patients and the CSF of patients with primary or metastatic CNS tumors. Pathway analysis revealed alterations in various metabolic pathways (e.g., glycine, choline and methionine degradation, dipthamide biosynthesis and glycolysis pathways, among others) between IDH-mutant and IDH-wildtype gliomas. Moreover, patients with IDH-mutant gliomas demonstrated higher levels of D-2-hydroxyglutarate in the CSF, in comparison to patients with other tumor types, or controls. This study demonstrates that analysis of CSF metabolites can be a clinically useful tool for diagnosing and monitoring patients with primary or metastatic CNS tumors.

**Electronic supplementary material:**

The online version of this article (10.1186/s40478-018-0588-z) contains supplementary material, which is available to authorized users.

## Introduction

Alterations in cellular metabolism are a critical part of cancer cell biology [8]. Studies of cellular metabolism have shown a variety of metabolic alterations in cancer [5, 9, 15, 22]. In the presence of oxygen, energy production in normal cells occurs primarily through oxidative phosphorylation. In contrast, anaerobic glycolysis followed by lactic acid fermentation, is utilized to produce energy in the absence of oxygen. However, many cancer cells produce energy through glycolysis and lactic acid fermentation, even in the presence of oxygen, a phenomenon called the Warburg effect [11]. Metabolic alterations in cancer cells can be useful in the diagnosis and monitoring of cancer patients. For example, the Warburg effect leads to an increased rate of glycolysis that is accompanied by an increase in glucose uptake, this becomes the basis for the use of fluorodeoxyglucose as a tracer for positron emission tomography (PET) studies [14].

Mutations in genes involved in important metabolic pathways, such as isocitrate dehydrogenase 1 and 2 (*IDH1/IDH2*)*,* are important cancer drivers (e.g., gliomas and leukemias) [19]. *IDH1/IDH2* mutations are associated with the production of an oncogenic metabolite, D-2-hydroxyglutarate (D-2-HG), which appears to be a critical aspect of tumor development [5, 21]. Increased levels of D-2-HG have been demonstrated in *IDH1/IDH2* mutant cells and culture media [5]. The survival of patients with IDH-mutant gliomas is significantly better than that of patients with IDH-wildtype gliomas. As a result, the WHO classification for central nervous system tumors was recently modified to include mutations in *IDH1/IDH2* as a critical part of the diagnosis of infiltrating gliomas [13]. Also, genes frequently mutated in CNS tumors (e.g., *PTEN*, *PI3K*) have known effects in metabolic pathways. For example, activation of the PI3K/AKT/mTOR pathway leads to increased translation of the hypoxia inducible factor 1α (HIF1α), increased glucose uptake, and increased uptake of essential amino acids [7]. Similarly, the transcription factor Myc can increase the expression of many metabolic enzymes [7].

The tools currently utilized for the diagnosis and monitoring of patients with CNS tumors include CNS imaging, evaluation of tumor cells in the cerebrospinal fluid (CSF-cytology) and brain biopsies. However, CNS imaging studies lack specificity, CSF-cytology has extremely poor sensitivity, and brain biopsies are an invasive procedure. Therefore, there is a critical need for more specific and less invasive methods for diagnosing and monitoring patients with CNS tumors. In particular, minimally invasive methods that inform aspects of CNS tumor biology that influence treatment decisions. Although it is recognized that metabolic alterations are common in cancer cells, it remains unclear to what extent the analysis of cellular metabolites in biofluids can be utilized in the clinical management of cancer patients. Several studies have demonstrated differences in circulating metabolites in the blood of patients with a variety of cancer types [3, 20]. However, blood is not an ideal fluid for detecting biomarkers in patients with CNS tumors [2, 6]. In contrast, studies have shown that the cerebrospinal fluid (CSF) is a better source of CNS-tumor-derived biomarkers [6, 10, 17]. In fact, elevated levels of D-2-HG have been demonstrated in the CSF of patients with IDH-mutant gliomas [10]. Differences in the levels of citric acid and lactic acid in the CSF of gliomas of different histologic grade have also been shown [16].

Considering the preliminary evidence showing alterations in metabolites in CNS tumors [10, 12] we decided to perform a comprehensive analysis of 129 metabolites in the CSF of patients with a variety of CNS tumor types. We analyzed the levels of metabolites in the context of CNS imaging and CSF-cytology results, routine clinical assays performed in the evaluation of patients with CNS malignancies. Our results provide insight into metabolic pathways that are altered in IDH-mutant gliomas in comparison to IDH-wildtype gliomas. Also, our data demonstrates elevated levels of D-2-HG in the CSF of patients with IDH-mutant gliomas. In summary, our data supports the idea that analysis of metabolites in the CSF can help in the diagnosis and monitoring of patients with CNS tumors.

## Methods

### Patients

**Fig. 1:**
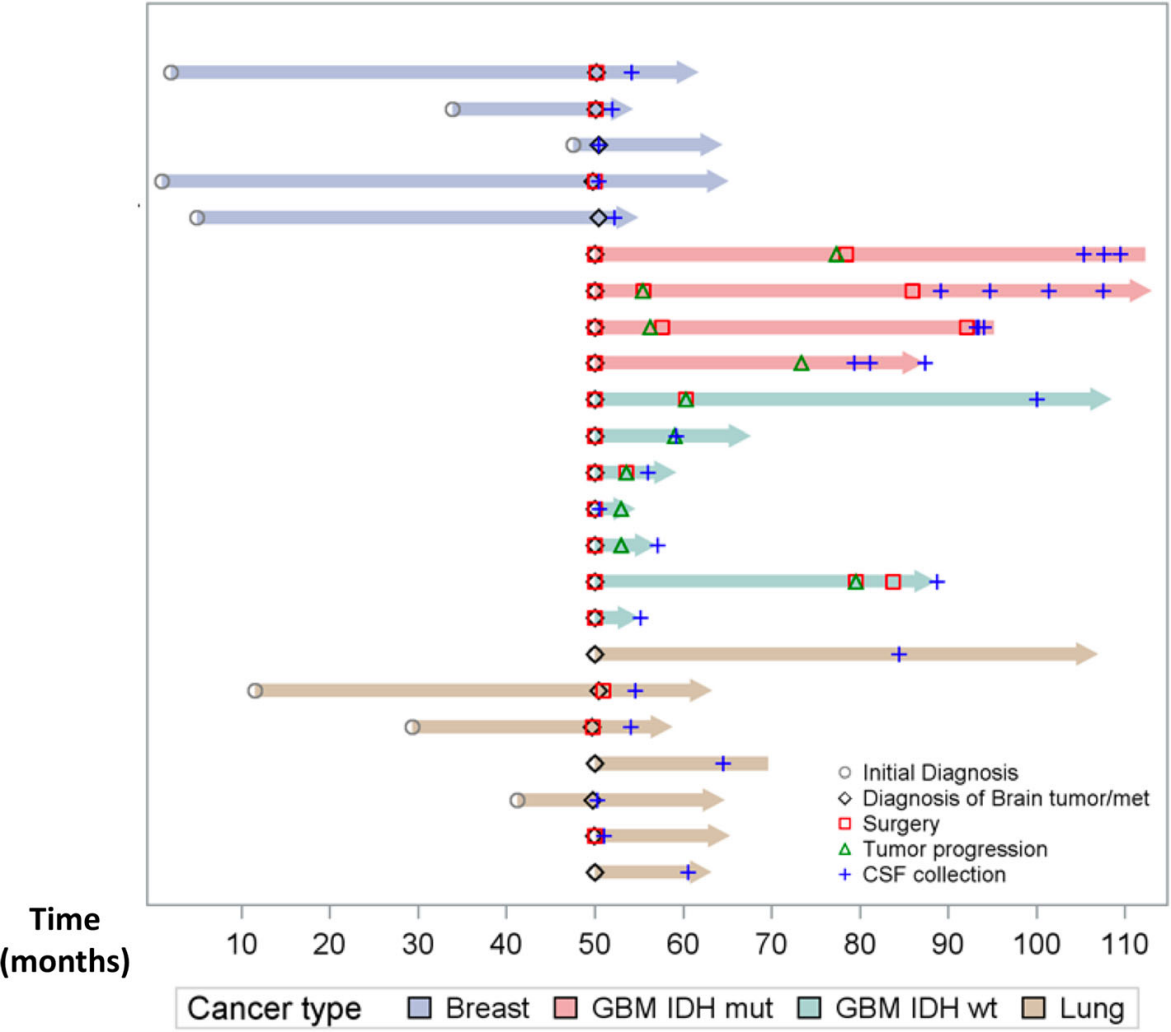
Swimmer plot depicting CSF sample collections for each patient. Each bar represents one subject included in the study. The blue crosses represent CSF collections. Surgery is indicated by the red square. Grey circles represent the initial diagnosis. The X-axis represents months. For some patients with metastatic disease (i.e., breast and lung cancer) the initial diagnosis of the systemic malignancy occured many months before the development of CNS disease

The study was approved by the institutional review board (IRB). All patients provided informed consent for participation of their samples in research. CSF was collected via lumbar puncture (LP) or intraventricular catheter (Ommaya reservoir, OM). Samples from patients with glioblastoma IDH-WT (*n* = 7), IDH-mutant (*n* = 4; 13 samples), metastatic lung cancer (n = 7) or metastatic breast cancer (*n* = 5) to the CNS were included (Table 1). All CSF samples from patients with CNS tumors were acquired during the course of the patient’s treatment (Fig. 1). Samples from patients with no history of cancer were included as controls (*n* = 8). All gliomas were sequenced for mutations in *IDH1* or *IDH2*. Contrast-enhanced brain MR with optimum 2D/3D images matching the CSF collection date were available for 23 of the 31 patients. MRI scans were interpreted as positive or negative for tumor by a neuroradiologist. Patient characteristics are included in Table 1.

**Table 1:** Patient characteristics

Patient ID	Age at time of CSF collection	LP vs OM	Sex	Race	Diagnosis	CSF cytology	MRI
1	42	LP	F	Unk	Chiari I malformation	N/A	Neg
2	47	LP	F	W	Aneurysm	N/A	Neg
3	56	LP	F	Unk	Left trigeminal neuralgia.	N/A	Neg
4	37	LP	F	Unk	Benign cyst	N/A	Neg
5	56	LP	M	W	Motor neuron disease	Neg	Neg
6	52	LP	F	Unk	Hydrocephalus	Neg	Neg
7	36	LP	M	AA	Hydrocephalus	Neg	Neg
8	20	LP	F	Unk	Pseudotumor cerebri	Neg	Neg
9	37	LP	F	W	Glioblastoma,IDH-WT	Neg	Pos
10	56	LP	M	W	Glioblastoma,IDH-WT	Neg	N/A
11	77	LP	M	His	Glioblastoma,IDH-WT	N/A	N/A
12	61	LP	F	W	Glioblastoma,IDH-WT	Neg	Pos
13	50	LP	M	W	Glioblastoma,IDH-WT	Neg	Pos
14	47	LP	M	A	Glioblastoma,IDH-WT	N/A	N/A
15	54	LP	M	A	Glioblastoma,IDH-WT	N/A	N/A
16	76	LP	F	Unk	Metastatic Lung Cancer	Neg	N/A
17	77	LP	M	A	Metastatic Lung Cancer	rare atypical cells	Pos
18	60	LP	F	W	Metastatic Lung Cancer	Neg	Pos
19	63	LP	F	W	Metastatic Lung Cancer	Neg	Pos
20	70	LP	M	W	Metastatic Lung Cancer	Neg	Pos
21	60	LP	F	Unk	Metastatic Lung Cancer	Neg	Pos
22	61	LP	F	W	Metastatic Lung Cancer	Neg	N/A
23	59	OM	F	A	Metastatic breast cancer	suspcious for lobular breast CA	Neg
24	59	LP	F	His	Metastatic breast cancer	consistent with metastatic carcinoma	Neg
25	62	LP	F	Unk	Metastatic breast cancer	negative	Pos
26	38	LP	F	His	Metastatic breast cancer	negative	Pos
27	73	OM	F	W	Metastatic breast cancer	negative	Pos
28	28	LP	M	W	Glioblastoma,IDH-mutant	N/A	N/A
29–30		OM				N/A	N/A
31	56	LP	M	Unk	Glioblastoma,IDH-mutant	Neg	Pos
32–33		OM				Neg	Pos
34	32	LP	M	W	Glioblastoma,IDH-mutant	atypical cells present	Pos
35–37		OM				Neg	Pos
38	23	LP	M	W	Glioblastoma,IDH-mutant	Neg	Pos
39–40		OM				Neg	Pos

### CSF-cytology

CSF was collected via LP or OM. Samples were processed within 2 h from collection time, and centrifuged at 1000 g for 10 min at 4 °C. The cell pellet was discarded and the CSF supernatant was aliquoted and immediately stored at − 80 °C. For CSF-cytology examination the CSF was centrifuged at 1500 rpm for 5 min. The supernatant was discarded and the cell pellet was resuspended in RPMI. Two – six drops of fluid were pipetted into cytospin chambers and the chambers centrifuged at 700 rpm for 7 min into one albumin-covered slide. The slides were stained with Wright-Giemsa and examined by a board certified cytopathologist.

### Imaging

Contrast-enhanced brain MR with optimum 2D/3D images were available for 23 patients. The images were analyzed for the presence of tumor by a board certified neuroradiologist.

### Metabolomic analysis

Targeted Metabolic profiling by LC-MS Single Reaction Monitoring (SRM) was used to characterize metabolites. We measured the metabolites using three different chromatographic methods (Additional file 1: Supplementary methods A-C), in each method metabolites were normalized with the spiked internal standards and data were log2-transformed. For each metabolite in the normalized dataset, a two-tailed t-test was used to compare their expression levels between two groups and ANOVA was used to compare more than two groups for their expression levels. Differentially expressed metabolites were identified after adjusting *p*-values for multiple hypothesis testing using the Benjamini-Hochberg method [1] and a False Discovery Rate (FDR) of < 0.25. A hierarchical cluster of the differentially expressed metabolites was generated using the R statistical system (https://www.r-project.org/). We have identified 129 metabolites. AUC (area under the receiver operating characteristic curve) as well as its 95% confidence interval was evaluated by the “DeLong” method with “pROC” package in R (Version 3.4.2) computing environment. The data was log2-transformed and normalized with internal standards on a per-sample, per-method basis.

## Results

### Patients and clinical characteristics

CSF (*n* = 40) from 31 patients; 13 males and 18 females with age ranging from 20 to 77 years old were included. CSF-cytology results were available for 22/31 patients. One out of three (1/3) available CSF-cytology results from patients with an IDH-mutant glioma was reported as “atypical cells present”, 2/5 CSF-cytology results from patients with metastatic breast cancer were reported as “suspicious” or “consistent” with metastatic carcinoma and 1/7 CSF-cytology results from patients with metastatic lung cancer were reported as “atypical cells present”. CSF-cytology results were available for 3/7 patients with IDH-WT gliomas, the 3 samples were reported as negative for tumor cells. CSF-cytology results for 4/8 control patients were available and the result was negative for tumor cells. In total, there were 14 instances in which CSF samples were reported as negative for tumor cells, but the MRI results demonstrated the presence of tumor involving the CNS (Table 1).

### Altered metabolites in the CSF of patients with CNS tumors

**Fig. 2:**
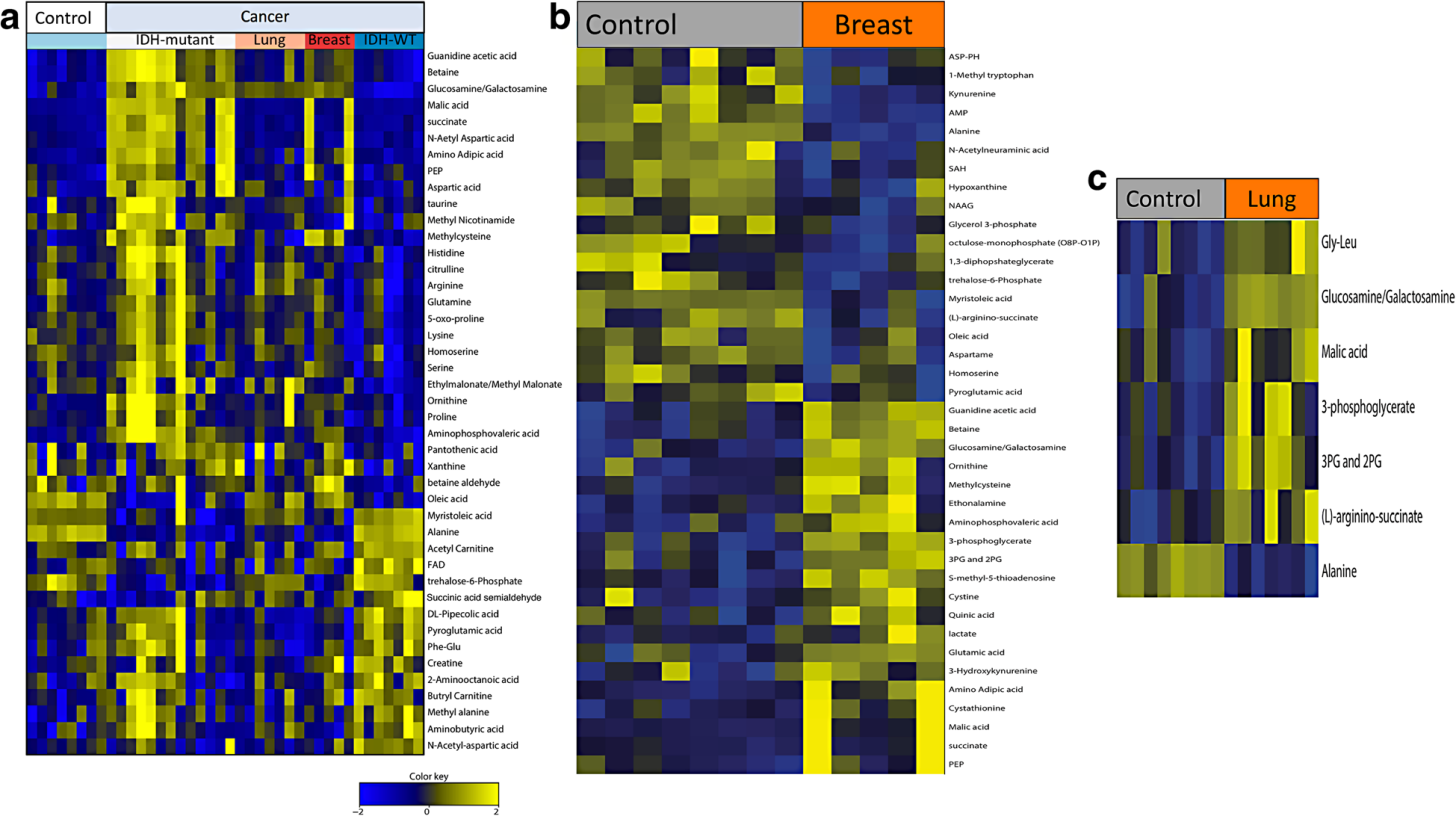
Analysis of metabolites distinguishes CSF from patients with CNS tumors from patients with non-neoplastic conditions. Heat map of unsupervised hierarchical clustering of metabolites showing metabolite levels in the CSF of patients with a variety of CNS tumors in comparison to control CSF obtained from patients with no history of cancer. **a** There are 43 differentially expressed metabolites between CSF from controls and CSF derived from patients with CNS tumors (IDH-mutant glioma, metastatic lung cancer or breast cancer to the CNS and IDH-WT gliomas). **b** We identified 20 metabolites (Guanidine acetic acid, betaine, glucosamine/galactosamine, ornithine, methylcysteine, ethonalamine, aminophosphovaleric acid, 3-phosphoglycerate, 3PG and 2PG, 5-methyl-5-thioadenosine, cysteine, quinic acid, lactate, glutamic acid, 3-hydroxykyurenine, amino adipic acid, cystathionine, malic acid, succinate, PEP) elevated in the CSF of patients with metastatic breast cancer to the CNS. **c** We identified 5 metabolites (Glycine/leucine, Glucosamine/galactosamine, Malic acid, 3-phosphoglycerate, (L)-arginino-succinate and Alanine) significantly elevated in the CSF of patients with metastatic lung cancer to the CNS

Using targeted metabolomics, we detected 129 named metabolites (in positive ionization and negative ionization mode) in the CSF of individuals with no cancer history. Differences in the abundance of 43 metabolites were found between CSF from control patients and CSF from patients with a history of a primary or metastatic CNS tumor. (Fig. 2a; FDR adjusted *p* < 0.25). By mapping the 43 altered metabolites into known metabolic pathways, we identified several pathways significantly affected, including glycine, arginine, choline, nitrogen metabolism and glycolysis (Additional file 2: Figure S1).

**Fig. 3:**
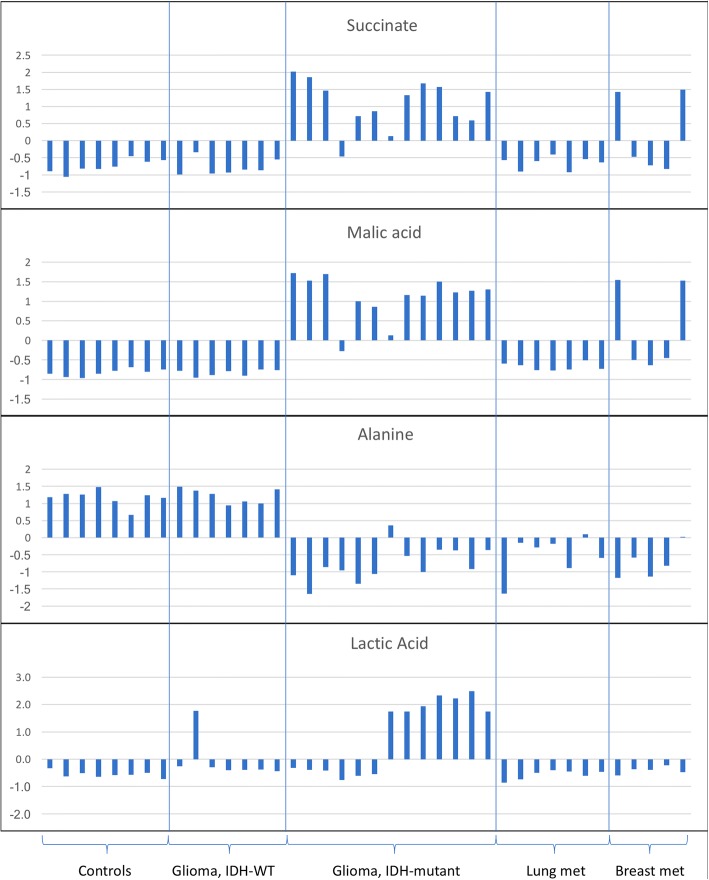
Differences in the levels of specific CSF metabolites. Differences in the levels of succinate, malic acid, alanine and lactic acid between 40 CSF samples from patients with no history of cancer (controls) or patients with gliomas IDH-WT, gliomas IDH-mutant, or metastatic lung or breast cancer to the CNS. Succinate and malic acid are elevated in all but one of the samples from patients with IDH-mutant glioma, in comparison to patients with IDH-WT gliomas, or controls. In contrast, alanine levels are reduced in CSF samples from patients with IDH-mutant gliomas or metastatic lung or breast carcinomas, in comparison to control CSF samples. Lactic acid levels were elevated in a subset of CSF samples from patients with IDH-mutant gliomas, in comparison to controls or patients with other cancer types. The Y-axis represents normalized log2 transformed values

**Fig. 4:**
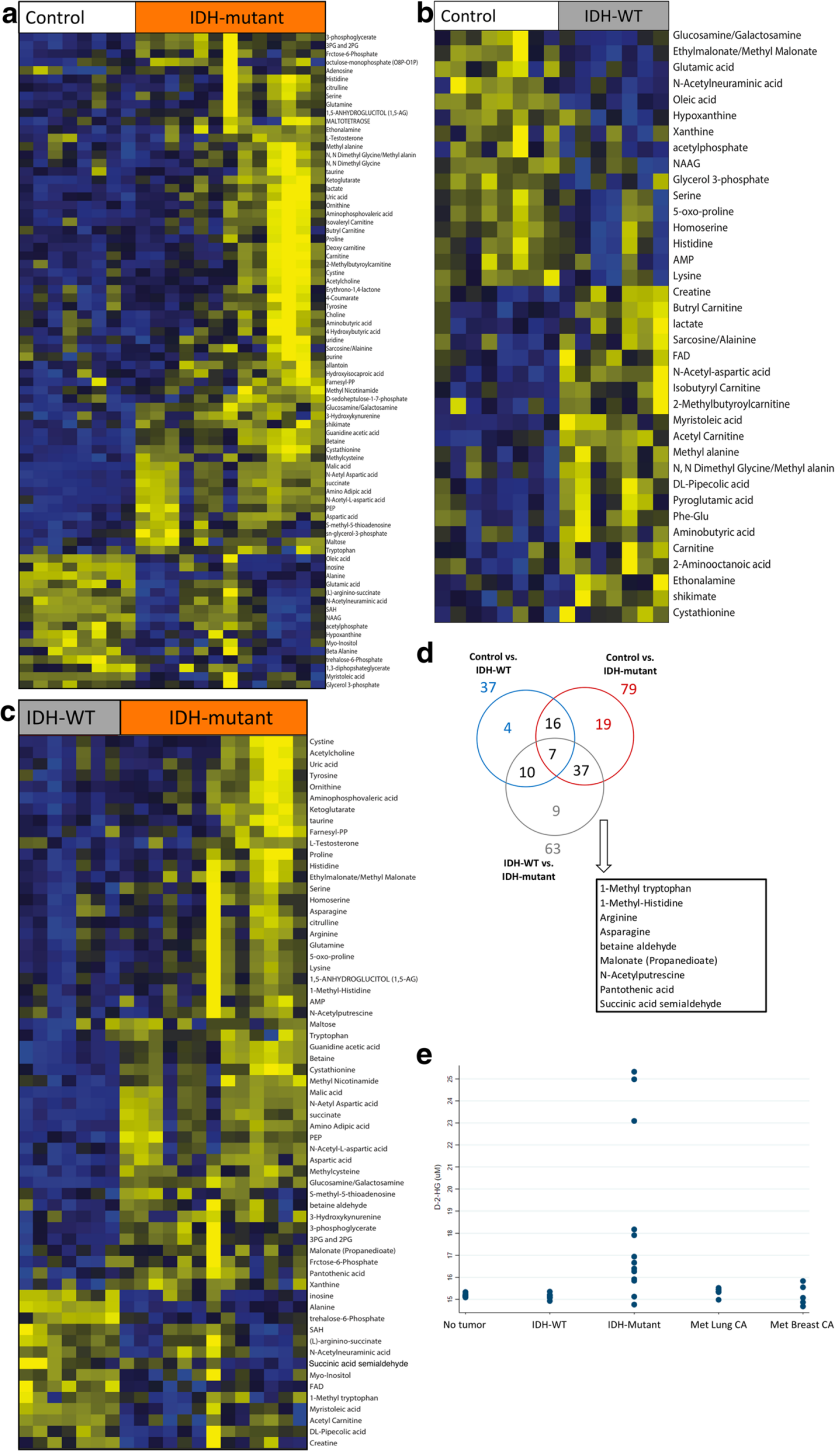
Analysis of metabolites in the CSF from IDH-mutant and IDH-WT gliomas. **a** Heat map showing differences in metabolites in the CSF of controls versus patients with IDH-mutant gliomas. **b** Heat map showing differences in CSF metabolites between controls and IDH-WT gliomas. **c** Heat map showing differences in CSF metabolites between IDH-mutant and IDH-WT gliomas. **d** Nine metabolites are significantly associated with the presence of the IDH-mutation in gliomas (1-Methyltryptophan, 1-Methyl-Histidine, Arginine, N-Acetylputrescine, Succinic acid semialdehyde, Malonate, betaine aldehyde, Pantothenic acid). **e** In addition, D-2-HG concentration is higher in the CSF of patients with IDH-mutant gliomas

A heat map depicting the unsupervised hierarchical clustering of samples is shown in Additional file 3: Figure S2. Tricarboxylic acid (TCA) cycle metabolites were found to be elevated in the CSF of patients with CNS tumors including malic acid and succinate. Succinate, malic acid and lactic acid were particularly elevated in IDH-mutant gliomas (Fig. 3). In addition, phosphoenolpyruvate (PEP) levels were elevated in the CSF of patients with IDH-mutant gliomas in comparison to patients with IDH-wildtype tumors (Fig. 4). We also found elevations in amino adipic acid in the CSF of patients with IDH-mutant gliomas. Acetylcarnitine and shikimate were elevated in the CSF of patients with IDH-WT gliomas in comparison to CSF from controls. In the case of patients with metastatic breast cancer, we identified the levels of 20 metabolites to be elevated in the CSF (Fig. 2b). Also, we identified 5 metabolites (Glycine/leucine, Glucosamine/galactosamine, Malic acid, 3-phosphoglycerate, (L)-arginino-succinate and Alanine) significantly elevated in the CSF of patients with metastatic lung cancer in comparison to CSF from controls (Fig. 2c).

### CSF metabolites in IDH-mutant versus IDH-WT gliomas

**Fig. 5:**
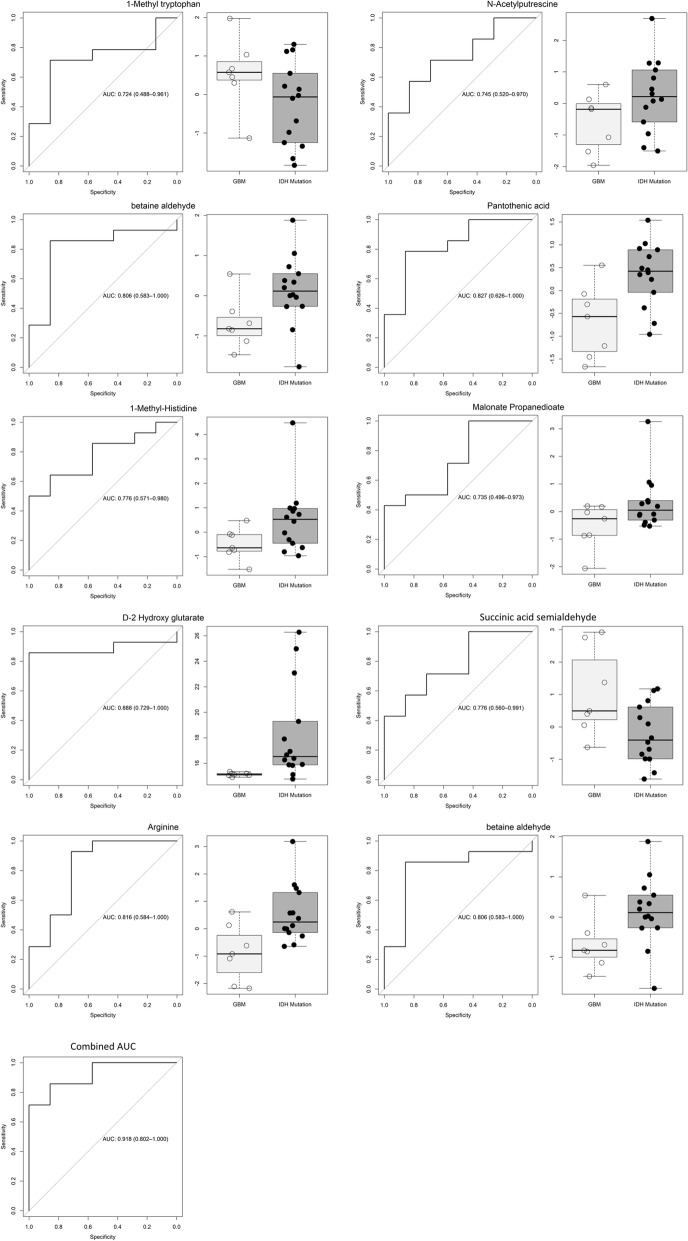
Area under the ROC curve. The levels of 10 metabolites are significantly different between IDH-mutant and IDH-WT gliomas. The area under the ROC curve for these metabolites individually and combined is shown. The area under the ROC curve for these 10 metabolites ranged from 0.724 to 0.888. In combination, the area under the ROC curve for the 10 metabolites was 0.918. The levels of these metabolites in CSF can help discriminate patients with IDH-mutant gliomas from IDH-WT gliomas

We identified 37 differential CSF metabolites between IDH-WT gliomas and controls, 79 differential metabolites between IDH-mutant gliomas and controls, and 63 differential metabolites between IDH-WT and IDH-mutant gliomas (Fig. 4). Further analysis identified several metabolites (1-methyl tryptophan, 1-methyl-histidine, arginine, asparagine, N-acetylputrescine, succinic acid semialdehyde, malonate, betaine aldehyde and pantothenic acid) that are associated with the presence of an *IDH1* mutation (Fig. 4d). In addition, we detected higher D-2HG levels in the CSF of patients with IDH-mutant gliomas (Fig. 4e). These metabolites were further analyzed individually for the area under the curve (AUC), in the receiver operator characteristics (ROC) curve, to evaluate the ability of each metabolite to discriminate IDH-mutant from IDH-WT gliomas. Individual metabolites were found to have a significant AUC between 0.724–0.888 (Fig. 5). Taken together, the 10 metabolites had a combined AUC of 0.918. While D-2-HG, malic acid and succinate levels were higher in IDH-mutant gliomas, the levels of alanine where significantly elevated in patients with IDH-WT gliomas compared to IDH-mutant tumors (Fig. 3).

### Lumbar puncture versus intraventricular catheter

We compared the levels of metabolites in CSF collected by lumbar puncture (LP) and CSF collected from an intraventricular catheter (Ommaya reservoir, OM). The was done by fitting a linear mixed effect model and conducting the likelihood ratio test. To account for the multiplicity, the *p* value was adjusted by Benjamini and Hochberg method [1]. An adjusted *p* value of less than 0.05 was considered statistically significant. We did not observe statistically significant differences in the levels of metabolites in samples obtained via LP versus OM (Table 1).

## Discussion

Our data shows that it is possible to discriminate CSF from patients with CNS tumors from CSF obtained from patients with non-neoplastic conditions. There are 14 CSF samples that were reported as negative for tumor by CSF-cytology, even when tumor was detected on the MRI, highlighting the limited sensitivity of CSF-cytology for detecting CNS malignancies, and the need for more sensitive and specific methodology to evaluate patients with CNS tumors. Only 1/12 (~ 8%) patients with a metastatic CNS tumor had a positive CSF-cytology result. Our results demonstrate that analysis of CSF metabolites could help identify patients with primary or metastatic CNS tumors, even in the absence of detectable tumor cells in the CSF.

Metabolites in nitrogen metabolism and aminoacyl-tRNA biosynthesis were elevated in the CSF of patients with CNS tumors. Also, glycine, serine threonine, alanine, aspartate and glutamate were present at significantly different levels in the CSF of patients with tumor when compared to controls. These data show that the metabolism of non-essential aminoacids is altered in the CSF of patients with brain tumors and elevations of these amino acids in the CSF could indicate a neoplastic process. Acetylcarnitine and shikimate were elevated in the CSF of patients with IDH-WT-gliomas in comparison to control CSF, an observation that is consistent with a previous report that analyzed CSF from 10 patients with gliomas [12].

It is not surprising that several of the altered metabolites are involved in the TCA cycle. Malic acid and succinate were particularly elevated in IDH-mutant gliomas, consistent with dysregulation of the TCA cycle in these tumors. These data indicate that TCA metabolite alterations in the CSF of patients with IDH-mutant gliomas could be helpful in distinguishing IDH-mutant from IDH-WT tumors. Also, our data shows higher D-2-HG levels in the CSF of patients with IDH-mutant gliomas, consistent with prior reports [5, 10, 18]. In addition, phosphoenolpyruvate (PEP) levels were elevated in the CSF of patients with IDH-mutant gliomas in comparison to patients with IDH-wildtype tumors. Elevated PEP levels have been previously described in glioblastoma tissue samples [4].

To our knowledge, this study is one of the most comprehensive analysis of CSF metabolites in patients with different types of primary or metastatic CNS tumors [12, 16, 23]. Our results show that it is possible to discriminate CSF from patients with IDH-mutant or IDH-WT gliomas and metastatic carcinomas, from patients with non-neoplastic conditions. One limitation of our study is that CSF samples were obtained during or after the patient’s treatment. Although the treatment for IDH-mutant and IDH-wildtype gliomas is similar, it is possible that some of the changes in metabolites in CSF could be influenced by the patient’s treatment. Therefore, additional studies with CSF obtained prior to therapeutic intervention will be greatly informative. It is important to highlight that metabolomic analysis can be performed with ~100uL of CSF in less than 24 h, and our results provide evidence for tumor-specific metabolic signatures that can help in discriminating neoplastic from non-neoplastic disease. Although studies have postulated differences in CSF biomarkers associated with collection method [10], we did not observe statistically significant differences in the levels of metabolites in samples obtained via LP versus intraventricular catheter. These suggests that although the collection method might influence the levels of some metabolites, it does not have significant effect on the levels of all metabolites. In conclusion, our data suggest that metabolomic analysis of CSF can provide clinically useful information with a fast turn-around-time, which could be helpful in the evaluation of patients with CNS tumors. This method can serve to complement the measurements of other tumor biomarkers (e.g., circulating tumor DNA) and increase the sensitivity and specificity of CSF analysis as a liquid biopsy approach for patients with CNS malignancies.

## Additional files

Additional file 1: Supplementary methods. (PDF 167 kb)

Additional file 2: **Figure S1.** Pathway analysis was performed using ingenuity pathway analysis (IPA) through overlap statistics. In this study, metabolite with raw p value less than 0.05 and absolute fold change larger than 1.5 was considered as significant metabolites. Enrichment in the pathway was evaluated by Fisher’s Exact test. The pathways with adjusted p value using Benjamini and Hochberg method less than 0.01 were reported and generated in barplot. (TIFF 57607 kb)

Additional file 3: **Figure S2.** Heat map of unsupervised hierarchical clustering of samples. Unsupervised hierarchical clustering using the complete agglomeration method was used for metabolite and sample clustering. (TIFF 4103 kb)
