# Supplementary material for: Analysis of cerebrospinal fluid metabolites in patients with primary or metastatic central nervous system tumors
Source: Acta Neuropathol Commun. 2018 Aug 31;6:85. doi: 10.1186/s40478-018-0588-z (PMC6117959; doi:10.1186/s40478-018-0588-z)
Supplement: Supplementary file 1 — Supplementary methods. (PDF 167 kb) [file 40478_2018_588_MOESM1_ESM.pdf]

Supplementary methods

Metabolic analysis of CSF samples:

Metabolites were extracted from CSF, and CSF pool was used as quality controls following the extraction procedure. All the CSF samples used for this study were stored at -140°C. 100 ul of CSF was used for the metabolic extraction. The extraction step started with the addition of 750 µL ice-cold methanol:water (4:1) containing 20 µL spiked internal standards to each CSF sample. Ice-cold chloroform and water was added in a 3:1 ratio for a final proportion of 1:4:3:1 water:methanol:chloroform:water. The organic (methanol and chloroform) and aqueous layers were mixed, dried and resuspended with 50:50 methanol: water. The extract was deproteinized using a 3kDa molecular filter (Amicon ultracel-3K Membrane; Millipore Corporation, Billerica, MA) and the filtrate was dried under vacuum (Genevac EZ-2plus; Gardiner, Stone Ridge, NY). Prior to mass spectrometry, the dried extracts were re-suspended in identical volumes of injection solvent composed of 1:1 water: methanol and were subjected to liquid chromatography-mass spectrometry.

Liquid Chromatography-Mass spectrometry (LC-MS)

Extracted CSF samples were injected and analyzed using a 6490 triple quadrupole mass spectrometer (Agilent Technologies, Santa Clara, CA) coupled to a HPLC system (Agilent Technologies, Santa Clara, CA) via single reaction monitoring (SRM). A total number of ~129 endogenous metabolites were chosen due to their involvement in central pathways and in a number of other biological functions. Source parameters were as follows: Gas temperature- 250 °C; Gas flow- 14 l/min; Nebulizer - 20psi; Sheath gas temperature - 350 °C; Sheath gas flow- 12 l/min; Capillary - 3000 V positive and 3000 V negative; Nozzle voltage- 1500 V positive and 1500 V negative. Approximately 8–11 data points were acquired per detected metabolite.

We used different methods to measure the ~129 metabolites as follows:

Method A: ESI positive mode was used in method A. The HPLC column was Waters XBridge Amide 3.5 µm, 4.6 x 100 mm. Mobile phase A and B were 0.1% formic acid in water and acetonitrile respectively. Gradient: 0 min-85 % B; 3-12 min- 85% to 10 % B, 12-15 min-10 % B, 16 min- 85% -B, followed by re-equilibration end of the gradient- the 23 min to the initial starting condition 85% B. Flow rate: 0.3 ml/min. Injection Volume 5ul.

Method B: ESI negative mode was used in method B. The HPLC column was Waters XBridge

Amide 3.5 µm, 4.6 x 100 mm. Mobile phase A and B were 20 mM ammonium acetate in water with pH 9.0 and 100% acetonitrile respectively. Gradient: 0 min-85 % B; 0-3 min- 85 % to 30% B, 3-12 min-30%-2 % B, 12-15 min- 2% -B, 15-16 min- 85% B followed by re-equilibration end of the gradient- the 23rd min to the initial starting condition 85% B. Flow rate: 0.3 ml/min. Injection Volume 10ul.

***Method C*:** ESI negative mode was used in method C. The HPLC column was Luna 3 µm NH2 100 A, 150 x 2 mm. Mobile phase A and B were 20 mM ammonium acetate in water with pH 9.0 and 100% acetonitrile respectively. Gradient: 0 min-85 % B; 0-3 min- 85 % to 30% B, 3-12 min-30%-2 % B, 12-15 min- 2% -B, 15-16 min- 85% B followed by re-equilibration end of the gradient- the 23rd min to the initial starting condition 85% B. Flow rate: 0.3 ml/min. Injection Volume 10ul.

***D-2-Hydroxyglutarate levels in CSF*:** The relative abundance of D- and L-2HG in CSF samples from patients with different tumor types was measured using chemical derivatization of 2HG as recently described (Struys et al., 2004). CSF (25 µL) spiked with internal standard (D-2-HG-d3) were extracted by protein precipitation with 50 µL 0.1M zinc sulphate and the addition of 300 µL methanol. The supernatant was then dried down, and 100 µL of 50 mg/mL DATAN in a 4:1 solution of dichloromethane and acetic acid was added. The samples were heated to 75 °C for 30 minutes and were allowed to cool before drying and reconstitution with 100 µL water.

Prepare stocked IS solution, deuterated (RS)- 2-hydroxyglutaric acid, disodium salt (2,2,2-D3; OD) 95%. Concentration is 50 ug/ml in water, Prepare DATAN solution, 50 mg/ml in methylene chloride-acetic acid (4:1, v/v). 50 ul of internal standard working solution was added to each sample vortex each sample for 5 min add 450 ul of Choloroform and vortex for 2 min, incubate at -20°C for 20 min and centrifuge for 10 min. Take both layers and dry 100 ul of freshly prepared DATAN solution was added on the dry residue Incubate at 80 degrees for 40 min Cooling to room temp, evaporated to dryness the dry residue was dissolved in 200 ul of a mixture containing 2 mM aqueous ammonium formate pH3.1 solution. 20 ul of sample were injected for analysis.

Using a Agilent 6490 QQQ MS, 10 µL of sample was injected onto a agilent XDB eclipse -C18, 4.6X150 mm column. A gradient elution was used with initial conditions of 95% mobile phase A (1.5 mM ammonium formate in water adjusted to pH 3.6 with formic acid) and 5% mobile phase B (methanol) for 5 minutes, stepping up to 30% mobile phase B for 1.5 minutes, before returning to initial conditions for 2.1 minutes (total run time was 8.6 minutes Agilent 6490 QQQ MS tandem mass-spectrometer was used and the specific transitions monitored were m/z 363>147 (L- and D-2-HG) and 366>150 (L- and D-2- HG-d3).
