# Supplementary figures and images for: Analysis of cerebrospinal fluid metabolites in patients with primary or metastatic central nervous system tumors
Source: Acta Neuropathol Commun. 2018 Aug 31;6:85. doi: 10.1186/s40478-018-0588-z (PMC6117959; doi:10.1186/s40478-018-0588-z)

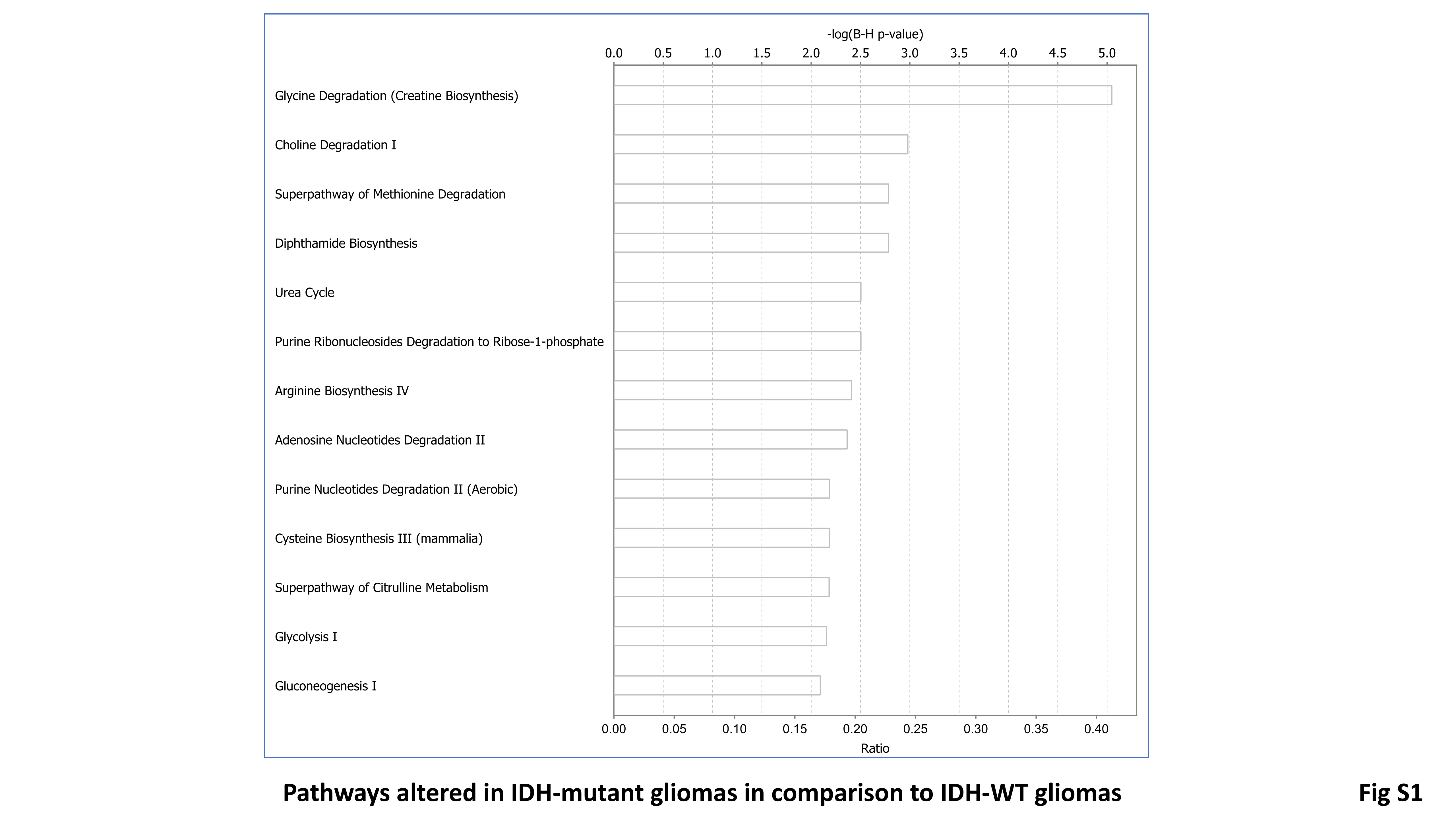

Supplement: Supplementary file 2 — Figure S1. Pathway analysis was performed using ingenuity pathway analysis (IPA) through overlap statistics. In this study, metabolite with raw p value less than 0.05 and absolute fold change larger than 1.5 was considered as significant metabolites. Enrichment in the pathway was evaluated by Fisher’s Exact test. The pathways with adjusted p value using Benjamini and Hochberg method less than 0.01 were reported and generated in barplot. (TIFF 57607 kb) [file 40478_2018_588_MOESM2_ESM.tiff]

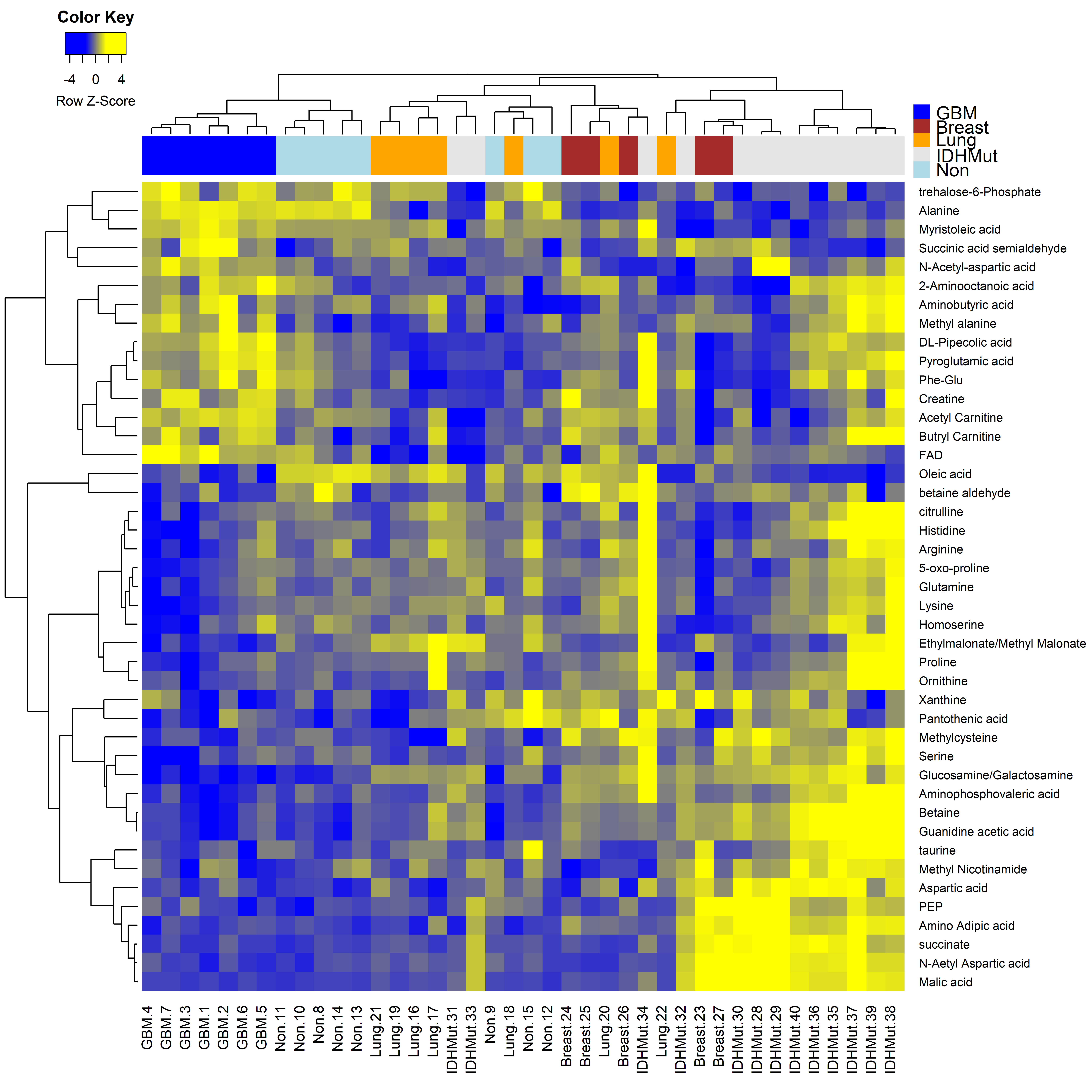

Supplement: Supplementary file 3 — Figure S2. Heat map of unsupervised hierarchical clustering of samples. Unsupervised hierarchical clustering using the complete agglomeration method was used for metabolite and sample clustering. (TIFF 4103 kb) [file 40478_2018_588_MOESM3_ESM.tiff]
